# Supplementary figures and images for: A Reassessment of Positive Growth Effects of Expressed Random Sequence Clones in E. coli Suggests Direct Adaptive Functions
Source: Genome Biol Evol. 2026 Jun 26;18(7):evag159. doi: 10.1093/gbe/evag159 (PMC13361975; doi:10.1093/gbe/evag159)

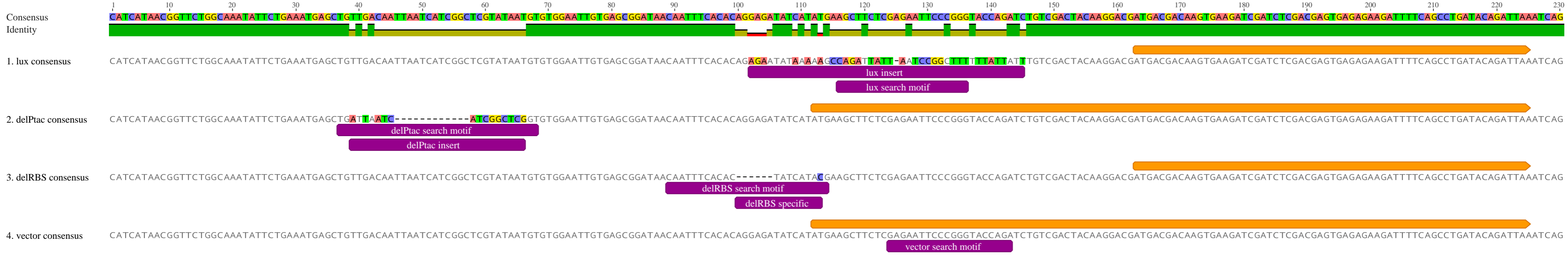

Supplement: evag159_Supplementary_Data [file evag159_supplementary_data.zip › supplFigureS5_vector_constructs.pdf]
